# Associations between Parental Social Support and Early Childhood Screen-time in US Children

**DOI:** 10.21203/rs.3.rs-6822093/v1

**Published:** 2025-06-25

**Authors:** Elise Warda, Suzy Tomopoulos, Michelle Katzow, Nikita Nagpal, Carol Duh-Leong

**Affiliations:** New York University; New York University; Northwell Health; New York University; New York University

**Keywords:** social support, family health, community health, parenting, screen-time

## Abstract

Excessive childhood screen-time is associated with adverse lifestyle and mental health outcomes. Studies suggest that social support, perceived assistance from others, can empower parents to manage screen-time; however, it is unclear which sources of support are relevant and whether income influences these associations. We examined whether individual, family, and neighborhood social support are associated with optimal early childhood screen-time. We performed a cross-sectional analysis of children aged 0–5 years from the 2022 National Survey of Children’s Health. Using American Academy of Pediatrics recommendations, we defined optimal screen-time as one hour or less daily. Support was self-reported by parents and included: 1) individual (day-to-day help), 2) family (family cohesion), 3) neighborhood (community support), and 4) cumulative (count 0–3 of all sources). We performed regression analyses, adjusting for race, ethnicity, maternal age, education, and income, with stratification by income. Among 19,741 children (51% male, 65% Non-Hispanic White, 55.9% low-income), 29.9% had optimal screen-time. Individual (OR 1.34, 95% CI, 1.11–1.62), family (OR 1.37, 95% CI, 1.10–1.71), neighborhood (OR 1.16, 95% CI, 1.07–1.38), and cumulative support (OR 2.24, 95% CI, 1.37–3.67) were associated with optimal screen-time. Individual support was associated with optimal screen-time in higher-income families (OR 2.12, 95% CI, 1.61–2.79). Family (OR 1.39, 95% CI, 1.05–1.88), neighborhood (OR 1.45, 95% CI, 1.17–1.66), and cumulative support (OR 2.46, 95% CI, 1.39–4.33) were associated with optimal screen-time in low-income families. All sources of social support are associated with optimal screen-time, but associations vary by income. Strengthening support may promote healthier screen-time, particularly in low-income families.

## Introduction

Screen-time—time spent in front of a television, tablet, phone, or computer for entertainment or social media – is steadily increasing. In children under the age of eight, average time spent using mobile media increased nine-fold from five minutes in 2011 to 48 minutes in 2017 (Rideout, 2017). Excessive screen-time affects eating patterns, exercise, and mental wellbeing, and thus screen-time has been associated with a myriad of adverse cognitive and cardiometabolic outcomes across childhood and throughout life including depressive symptoms, poorer executive function, sleep problems, and obesity (Kaur et al., 2019; Lin et al., 1979; Sisson et al., 2009; Stiglic & Viner, 2019; Wolf et al., 2018). The literature has consistently shown differences in screen time across race, ethnicity, and income (Hoyos Cillero & Jago, 2010; Lin et al., 1979). Given that screen-time is strongly associated with adverse outcomes, many studies have attempted to identify other factors influencing differences in screen time in childhood to identify possible avenues for intervention.

Early childhood is crucial for socioemotional, behavioral, and physical development, making screen-time in this age group particularly important, as habits established in early childhood are consistent with those seen in later childhood and adolescence (Anderson et al., 2003; Dumuid, 2020). Currently, the American Academy of Pediatrics (AAP) recommends zero hours of screen-time for children under two (aside from social video calls) and one hour or less daily for children aged two to five (Hill et al., 2016). While risk factors for excessive screen-time—such as low socioeconomic status and high parental screen-time—are well documented, less is known about protective factors promoting optimal screen-time (Sisson et al., 2009; Xu et al., 2015).

Parental factors are key in early childhood behavior due to the dependency of young children’s routines on caregivers (Caring Relationships, 2017). Parenting stress, defined as an adverse psychological response to the demands of raising children, is known to be associated with screen-time and other sedentary behaviors (Bornstein, M. H., 2002; McDaniel & Radesky, 2020). As a widespread concern, the US Surgeon General’s 2024 advisory encourages addressing parental stress, mental health, and well-being in order to improve parent and child overall health (Murthy, 2024). Research studying low-income families has shown that parental stress and social isolation contribute to excessive childhood screen-time (Hoyos Cillero & Jago, 2010; Lampard et al., 2013; Tooth et al., 2021). Parenting stress may increase the likelihood that screen-time is used as a coping mechanism, given that screen-time provides a safe, low-cost form of distraction that frees up time for parents (Auxier et al., 2020; Morawska et al., 2023; Stress in America, 2023).

**Figure 1:**
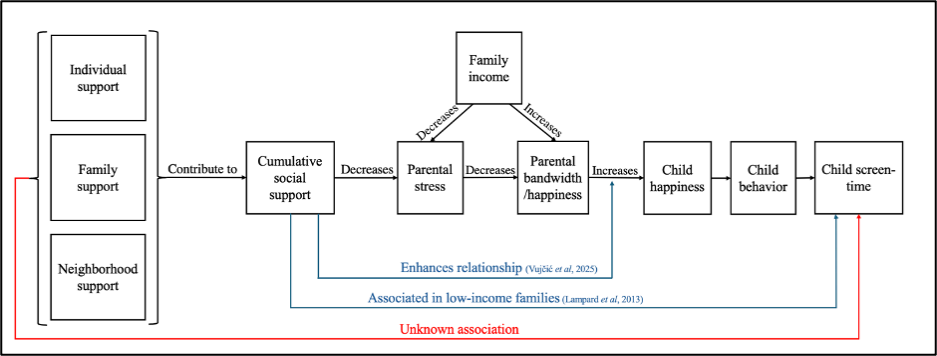
Proposed concept diagram for parental support, stress, and child behavior. Hypothesized associations of social supports from individual, family, and neighborhood sources with childhood screen-time may be mediated through parental factors including stress, bandwidth, and income.

Social support is defined as perceived care or assistance accessible through social ties and is linked to improved physical and mental health in children and adults (Lin et al., 1979; Martino et al., 2015). Social support appears to have positive effects on the entire family, as current literature shows that while parents’ happiness is associated with their child’s happiness, having social support strengthens this relationship, leading to positive emotional effects on the entire family unit (Vujčić et al., 2025). Previous research has shown that generalized social support, not accounting for specific sources of support, is associated with lower early childhood screen-time in children from low-income families, which may be explained by the concept of parental stress (Fig. 1; Lampard et al., 2013; Murthy, 2024).

Though generalized social support is associated with screen-time (Lampard et al., 2013), it is still unclear which specific sources of social support—individual, family, or neighborhood—are protective against screen-time in early childhood. To address these gaps, we conducted a secondary analysis of a nationally representative sample of US children aged 0–5 years. We hypothesized that parental individual, family, and neighborhood support would be associated with lower early childhood screen-time. Given the increased stress and social isolation seen in poverty, we hypothesized that associations would vary with income.

## Methods

### Data collection

We performed a secondary analysis utilizing data on screen-time and social support from the 2022 National Survey of Children’s Health (NSCH) (*National Survey of Children’s Health, Stata Indicator Dataset.* 2022). The NSCH is a population-based survey representative of US children that administers questionnaires online and by mail to randomly selected parents living in the United States about their children’s health (*National Survey of Children’s Health, Sampling and Survey Administration*, 2022; United States Census Bureau, 2024). We analyzed data from parents of children aged 0–5 to represent early childhood prior to school entry. This study was exempted from review by the [institution name] Institutional Review Board because the NSCH dataset is a publicly available de-identified dataset and thus not human subject research.

#### Outcome: Screen-time

The NSCH defined screen-time as time spent using an electronic device with a screen for entertainment. The question was, “On most weekdays, about how much time did this child spend in front of a TV, computer, cellphone or other electronic device watching programs, playing games, accessing the internet or using social media? (Do not include time spent doing schoolwork.)” Parents could answer “Less than 1 hour,” “1 hour,” “2 hours,” “3 hours,” or “4 or more hours” (United States Census Bureau, 2024). Using AAP guidelines from 2016, we established our screen-time outcome, optimal screen-time, as equal to or less than one hour of screen-time daily for this age group (Hill et al., 2016).

### Predictors: Individual, Family, Neighborhood, and Cumulative Social Support

The NSCH assessed individual support by asking parents to answer the question “During the past 12 months, was there someone that you could turn to for day-to-day emotional support with parenting or raising children?” Parents could answer “Yes” or “No” (United States Census Bureau, 2024). Family support was assessed as a composite measure based on parental responses to the following questions: “When your family faces problems, how often are you likely to do each of the following? (a) Talk together about what to do, (b) Work together to solve our problems, (c) Know we have strengths to draw on, and (d) Stay hopeful even in difficult times.” Parents could respond none of the time, some of the time, most of the time, or all of the time to each item. Based on how other studies have dichotomized this variable, we defined having family support as answering all or most of the time to all four items (Westphaln et al., 2022). We defined neighborhood support using the NSCH composite measure including the prompts, “People in this neighborhood help each other out,” “We watch out for each other’s children in this neighborhood,” and “When we encounter difficulties, we know where to go for help in our community.” Parents could answer that they definitely agree, somewhat agree, somewhat disagree, or definitely disagree to each prompt. The NSCH defined having support as responding somewhat agree or definitely agree to at least one prompt (Evans et al., 2011). We created the cumulative support variable by summing the presence of all three support types (individual, family, and neighborhood), assigning a score of 0–3.

#### Covariates:

Potential confounding measures were selected based on their association with screen-time in previous research. These included race, ethnicity, maternal age, parental education, and parental income (Hoyos Cillero & Jago, 2010; Lampard et al., 2013; Martino et al., 2015; Xu et al., 2015). The NSCH assessed race and ethnicity as one of the following categories: Hispanic, White non-Hispanic, Black non-Hispanic, and Other/Multi-racial non-Hispanic. Maternal age was self-reported in years. We defined parental education as the highest level of education attained from one of four options: less than high school, high school graduate or GED, some college or technical school, or college degree or higher. We defined family income using household federal poverty level (FPL) income groups from the year 2021. The NSCH does not directly measure parenting stress but does look at aggravation with the question “Does this child have parents who felt aggravated by parenting during the past month?” Given prior research linking parental stress, screen time, and income, we adjusted for parental aggravation to isolate potential effects of social support beyond decreasing stress alone (McDaniel & Radesky, 2020). Parents could answer that they “seldom [felt] aggravation” or “usually/always [felt] aggravation” (United States Census Bureau, 2024). We defined parental aggravation as present if parents reported that they usually/always felt aggravation, and absent if they seldom felt aggravation.

### Statistical Analysis:

First, we summarized our sample using weighted descriptive statistics. Then we used chi square models to assess bivariate associations between screen-time and individual, family, neighborhood, and cumulative support. We also used chi square models to evaluate the association between screen-time and parental aggravation. We then used logistic regression to model the relationship between optimal screen-time and each type of support in unadjusted and adjusted models for race, ethnicity, maternal age, parental education, and parental income. For any significant relationships between screen-time and each source of social support, we adjusted for parental aggravation in addition to the other possible confounders. We then ran the same set of regression models stratified by income level. To dichotomize the family income variable, low-income families were defined as those below 400% federal poverty level (FPL), and higher-income families were defined as those at or above 400% FPL (Larson & Halfon, 2010). Finally, we assessed moderation in separate unadjusted and adjusted logistic regression models, which separately included an income-by-support interaction term. All models were weighted for child-level measures to generalize for state and national child resident populations as described by the NSCH Methodology (United States Census Bureau, 2024).

## Results

Our sample included 19,741 children (weighted n = 21,470,296, Table 1). Of the sample, 10,081 children (weighted 51.07%) were male. The majority of the sample identified as White, non-Hispanic (weighted 64.78%), with 15.27% Hispanic, 5.07% Black, non-Hispanic children, and the remaining 14.88% identified as Other/Multi-racial, non-Hispanic. Among our sample, 5,894 (weighted 29.86%) children were reported to have optimal screen-time. There were 924 parents (weighted 4.81%) who reported experiencing parental aggravation (Table 1). For social support, 16,437 (weighted 83.26%) parents reported having individual support, 16,577 (weighted 87.80%) parents reported having family support, and 10,843 (weighted 54.92%) parents reported having neighborhood support (Table 1). There were 8,892 (weighted 47.69%) children in households whose parents had a score of three on cumulative support (Table 1).

**Figure 2:**
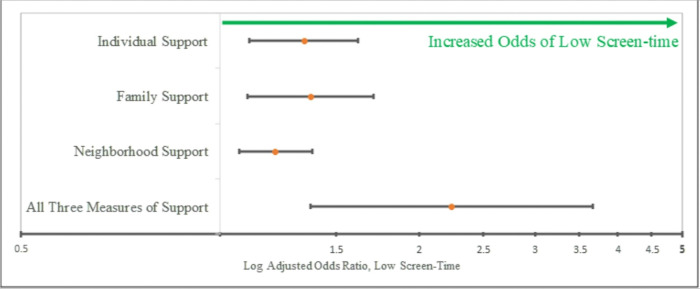
Adjusted Odds Ratio for Optimal Screen-time and Measures of Social Support. Parents were surveyed in the National Survey of Children’s Health whether they had individual support, family support, and/or neighborhood support and about their child’s screen-time. Weighted adjusted logistic regression models for parents of children aged 0–5 compared odds of low screen-time, one hour or less, in parents with or without sources of social support. Weighted adjusted and unadjusted logistic regression models for parents of children aged 0–5 also compared odds of low screen-time in parents with no types of social support versus cumulative support. Models were weighted for child-level measurements and adjusted for race/ethnicity, maternal age, parental education, and poverty level, (weighted n=21,470,296). Odds ratios were plotted on a log scale.

Individual support (OR 1.34, p = 0.003, Table 2), family support (OR 1.37, p = 0.006, Table 2)and neighborhood support (OR 1.17, p = 0.018, Table 2) were each associated with optimal early childhood screen-time even when adjusted for race, parental age, parental education, and parental income. These associations were also present in models that adjusted for parental aggravation (individual support OR 1.32, p = 0.005; family support OR 1.35, p = 0.008; neighborhood support OR 1.16; p = 0.026). Logistic regressions also revealed a significant association between a score of three for cumulative support and optimal screen-time which persisted even when adjusting for race, parental age, parental education, and parental income (OR 2.24, p = 0.001, Fig. 2).

**Figure 3:**
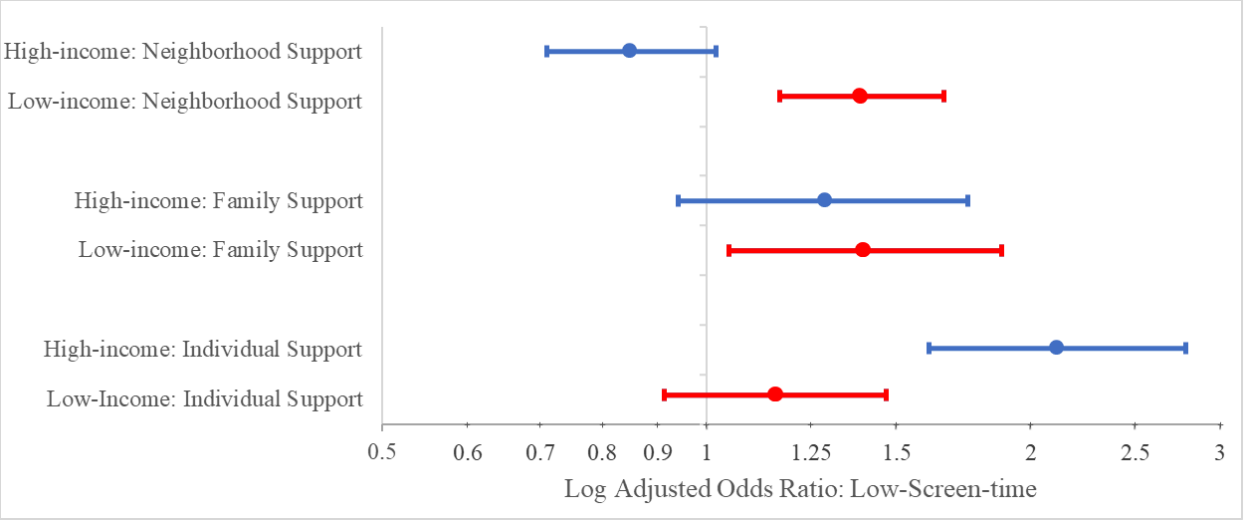
Adjusted Odds Ratio for Optimal Screen-time and Social Support, Stratified by Income. Weighted adjusted logistic regression models for families with and without individual, family, and neighborhood support were stratified by low vs higher income. Low-income families had an annual income of less than 400% federal poverty level, and higher-income families were those with an annual income at or above 400% federal poverty level. Models were weighted for child-level measurements and adjusted for race/ethnicity, maternal age, parental education, and poverty level. Odds ratios were plotted on a log scale.

Analyses stratified by income showed that among low-income families, neighborhood (OR 1.45, p < 0.001, Table 3), and family support (OR 1.39, p = 0.014, Table 3) were each independently associated with optimal screen-time in unadjusted models. The association with optimal screen-time was also present in low-income families between family support (OR 1.40, p = 0.022, Table 3) and with neighborhood support (OR 1.39, p < 0.001, Table 3) even when adjusting for covariates. These relationships were also significant in models adjusting for parental aggravation (family support OR 1.39, p = 0.028; neighborhood support OR 1.38, p < 0.001). However, individual support was not associated with screen-time among low-income families in unadjusted (OR 1.18, p = 0.130, Table 3) or adjusted models (OR 1.16, p = 0.228, Table 3). For higher-income families, individual support was associated with optimal screen-time in unadjusted (OR 2.12, p < 0.001, Table 3) and adjusted models (OR 2.12, p < 0.001, Table 3). In adjusted models accounting for parental aggravation, the relationship between individual support and screen-time in higher-income families was also significant (OR 2.09; p < 0.001). Neither neighborhood support (unadjusted OR 0.944, p = 0.528; adjusted OR 0.850, p = 0.073, Table 3) nor family support (unadjusted OR 1.35, p = 0.066; adjusted OR 1.29, p = 0.114; Table 3) was associated with optimal screen-time in higher-income families. For cumulative support, stratifying analysis by income revealed that cumulative support was associated with optimal screen-time in low-income-families (unadjusted OR 2.26, p = 0.001; adjusted OR 2.46, p = 0.002; Table 3) but not in higher-income-families (unadjusted OR 1.81, p = 0.107; adjusted OR 1.62, p = 0.256; Table 3). For income-level, the interaction term was statistically significant for individual support (unadjusted B = 1.29, p = 0.009; adjusted B = 1.27, p = 0.020) and neighborhood support (unadjusted B = 0.75, p < 0.001; adjusted B = 0.78, p = 0.000), but not for family support (unadjusted B = 0.99, p = 0.913; adjusted B = 0.99, p = 0.949; Fig. 3).

## Discussion

In a sample representative of US children aged 0–5, we detected associations between early childhood screen-time and parent perceived individual support, family support, neighborhood support, and cumulative support. Young children in families where parents reported having individual support with day-to-day parenting, family support in difficult scenarios, or neighborhood support from the community in times of need were more likely to have screen-time of less than one hour daily even when adjusting for socioeconomic confounders. Current recommendations for developing healthy childhood screen-use focus largely on parental education and establishing routines, but do not address social support (Hoyos Cillero & Jago, 2010). We also found that family income status moderates the relationships between individual and neighborhood support and screen-time.

We found that individual social support—day-to-day emotional support for parenting—was associated with optimal screen-time in US children aged 0–5. Prior research about social support and screen-time studied only generalized social support without specifying different sources (ex. individual, family, or neighborhood), so we contribute that support at the individual level is associated with optimal screen-time (Lampard et al., 2013). In the NSCH, individual support was a type of support specifically tied to parenting, which has not been previously studied in the context of screen-time. Parental engagement with their child’s media use has been shown to be protective against excessive screen-time, but parental bandwidth likely influences how often parents can monitor screen use (Lampard et al., 2013). Stressful life events can be buffered when mothers perceive having stronger personal support networks (Cochran et al, 1990), so perhaps by buffering stress, individual support enhances parental emotional and temporal bandwidth, enabling parents to monitor and engage with their child’s screen use. However, these associations held even when adjusting for parental aggravation, suggesting that other mechanisms are influencing these relationships. Perhaps individual support and screen-time are related through effects of individual support on parental mental health, or perhaps parents experiencing more stress display higher levels of screen-time themselves, which their children then learn. It has been shown that individual social support can assist families in various ways, (ie. providing emotional support, childcare, and/or financial resources) so future studies should examine whether different avenues of individual support (from a spouse, friend, family member, etc.) are most associated with screen-time to inform targeted interventions (Duh-Leong et al., 2021). Another explanation is that optimal childhood screen-use leads to improved parental individual social support is, as perhaps lower child screen-time frees up time for parents to build supportive relationships. Additionally, more studies are needed to better characterize the possible mechanisms by which individual support is associated with screen-time, including characterization of parental bandwidth, parental knowledge, and parental habits to better understand the directionality of this association and design targeted interventions to increase social support to address these specific factors.

We found that income moderated the relationship between individual support and screen-time, such that individual support was only associated with optimal screen-time among families with higher-income, but not in families with low-income. Prior research has focused on the relationship of generalized social support with screen-time among low-income families only (Lampard et al., 2013). It is possible that in low-income families with greater stress, parental bandwidth tends to fall below that of higher-income families, such that day-to-day support with parenting is insufficient to mitigate effects on screen-time. Similarly, perhaps for low-income families, having fewer available community resources, such as libraries or a safe park to play in, makes it so that daily parenting support is not impactful on screen-time because other options for entertainment are unavailable regardless. Future studies should also consider investigating the impact of other factors such as low health literacy, child complex healthcare needs, or child stress which may be more prevalent in lower income families and may impact both individual support and screen-time independently.

We identified that family support was associated with optimal screen-time in the full sample. Prior evidence has shown that generalized social support among low-income families supports healthy screen use in early childhood, so this study extends that evidence to show that family support is associated with screen-time in a wider population (Lampard et al., 2013). Our measure of family support from the NSCH assessed how well families solved problems and functioned cohesively. Research has shown that family functioning is linked to the development of obesity-related behaviors such as screen-time, consumption of sugary beverages, and eating processed foods, likely because cohesive families are better able to establish healthy routines and habits (Wen et al., 2011). Family cohesiveness also likely improves the family’s ability to deliver consistent messages about screen-time and therefore may enhance the child’s understanding of and adherence to rules about screen-time. Experts have recommended that families provide a consistent message and plan for child screen-time routines, so future studies ought to investigate the role of family closeness and cohesion in screen-time practices (American Academy of Pediatrics, 2024). Screen-time may also influence family support, for example if additional screen-time interferes with perceived or actual family connectedness. Future studies should investigate whether factors such as childhood routine stability or geographic distance between family members modulate the relationship between family support and early childhood screen-time.

For family support, we identified variation by income in stratified analysis, showing that family support was associated with optimal screen-time in low-income families, though the interaction term was not significant. Generalized support has been studied in low-income families only, and it had not been previously studied whether family support is associated with screen-time in low or higher-income families (Lampard et al., 2013). Therefore, we clarify that family support does appear to be associated with early childhood screen-time in low-income families, but not in higher-income families. One possible explanation is that higher-income families have the resources to develop healthy screen routines regardless of family cohesiveness. Higher-income families may have more support available from other avenues such that the presence of family support is less impactful on screen-time compared to low-income families. Low-income families may have routines that are more strongly affected by family cohesion, possibly due to cultural differences or living with a greater number of family members. Another explanation is that more screen-time enables connection with physically distant loved ones, and physical distance from family support may be impacted by income level. The lack of moderation by income suggests that intervention strategies focused on family support could be beneficial if implemented generally, or they may be most beneficial if directed to low-income families.

We identified that neighborhood support was associated with optimal screen-time in the general population. This extends prior evidence, which had studied generalized support alone, showing that support at the neighborhood level is associated with optimal screen-time in a wider population (Lampard et al., 2013). Parents who feel supported by their neighborhood networks may have more bandwidth for parenting activities and are likely better able to engage their children in other activities away from screens (such as reading, playing with toys, or going outdoors). More neighborhood support might also create the opportunity for parents to discuss and share general positive parenting practices, including but not limited to, the importance of monitoring screen-time in early childhood. As a result, knowledge sharing could both enhance parenting techniques and increase health literacy surrounding screen-time, empowering parents to interact with screens in a healthy manner. Future studies should consider evaluating the impact of covariates that characterize the community with measures such as neighborhood safety. One study showed that parents who perceive higher neighborhood safety tend to have children with lower screen-time who spend more time engaged in physical activity (Qu et al., 2023). Children in less safe neighborhoods may be encouraged to spend more time on a screen indoors, or they may use devices more often as a safety measure, such as by location sharing or frequent check-ins with parents.

We also found an association between neighborhood support and optimal screen-time in low-income families, but not in higher-income families. This extends prior evidence showing an association between generalized social support and screen-time in low-income populations to show that neighborhood sources contribute to this relationship (Lampard et al., 2013). In moderation, we found that income did impact the relationship of neighborhood support with screen-time. One possible explanation is that parents with low-income may have fewer community resources for alternative activities and therefore might utilize screens more often to keep their children occupied. Another explanation as mentioned previously is that neighborhood safety, which is likely inferior for low-income families, could contribute to higher screen utilization. Additionally, children with higher screen-time may have less time available for engaging in other community building activities with family, such that parents perceive or experience lower support. Intervention strategies on neighborhood support should be directed at low-income families and may be concurrently implemented with interventions that target family support, as similar groups may benefit from strengthening support of both levels.

We also found that parents who reported having greater cumulative support were more likely to have children who met screen-time recommendations compared to parents with fewer types of support. Additionally, in non-stratified analyses, our study showed that having cumulative support was independently associated with optimal screen-time even when adjusting for family income and other socio-economic factors including race/ethnicity, maternal age, and parental education level. In stratified analyses, cumulative support was associated with optimal screen-time in low-income families and was not associated with optimal screen-time in higher-income families. This supports prior evidence from studies that have looked broadly at social support in low-income families and found that generalized support is associated with screen-time in this population (Lampard et al., 2013). Higher-income families have not been the focus of prior studies, so these findings also extend the literature by demonstrating that generalized support may be most helpful for low-income families, or those who may be more stressed. It is likely that similar mechanisms driving the relationships between individual, family, and neighborhood support with screen-time are influencing cumulative support. It is also possible that, rather than social support driving optimal screen-time, optimal screen-time may influence social support. Perhaps screen-time in the form of device distraction reduces parental stress, allowing parents to expand their social networks in times of ease, thus having the largest effect on low-income households with higher stress.

We had hypothesized that the relationship between social support and screen-time was mediated largely by parental stress. Prior work has shown that parental stress was associated with both non-optimal screen-time and lower social support (McDaniel & Radesky, 2020; Shin et al., 2021). However, in our models evaluating the association of social support and screen-time, these relationships were still significant even when adjusting for parental aggravation. This shows that parental stress as measured in our study does not account for the entirety of the relationship between screen-time and social support. Perhaps other variables such as modeled parental screen-time, resource sharing in the form of books or toys, and/or child stress drive the relationship between social support and screen-time, with parental aggravation playing a smaller role than we had hypothesized. One other possibility is that the parental aggravation variable measured in the NSCH did not accurately characterize parental stress, which is multi-faceted and likely extends beyond aggravation. In our sample, less than 5% of parents reported parental stress (Table 1). With such a small proportion of families reporting aggravation, this suggests that the NSCH likely only identified parents experiencing the highest levels of parenting stress and did not account for mild and moderate levels of stress, which likely also contribute to screen-time. Future studies ought to characterize parenting stress more precisely with validated tools to evaluate the role of stress in the relationship between social support and screen-time.

We also found that non-optimal screen-time was highly prevalent in early childhood. Less than 30% of children in our sample met optimal screen-time recommendations of one hour or less daily (Table 1). This was consistent with prior studies that utilized NSCH data from 2018–2020, which found that 70.8% of children aged 0–5 had excessive screen-time of more than one hour daily (Qu et al., 2023). Recently, discourse surrounding screen-time has moved towards recognizing that families have different values and preferences about technology, thus encouraging parental monitoring of screen-time. Instead of strict numerical cut-offs, many are moving towards emphasizing parental co-engagement with screens and screen use for healthy purposes, such as for education or for social connection with family. This offers an important flexibility for families especially as the role of technology becomes more ingrained in everyday life. Given that most children in the United States have high amounts of screen-time, it is clear that many families have the potential to benefit if factors affecting screen use are better understood. In light of our findings, low-income families may have the most potential to benefit, especially with regard to family and neighborhood support. When counseling on screen-time, pediatricians can weave in the importance of social connection, which offers a myriad of physical and mental health benefits (Auxier et al., 2020; Qu et al., 2023).

Overall, we shed light on which levels of social support are related to screen-time by showing that individual, family, and neighborhood levels of support are independently associated with optimal screen-time in a widely representative sample. Our findings corroborate what has been demonstrated in prior studies, showing that in low-income families, having cumulative support was associated with optimal screen-time. We also showed that all types of social support were associated with optimal screen-time, but that the relationships with specific types of support and screen-time were modulated by family income. Generally, we suggest that multiple levels of social support can contribute to healthy screen-time in a more widely representative sample compared to prior studies and provides more detail about which families appear to be most affected by specific types of social support.

We analyzed data from a broadly representative sample of young children using the NSCH, but therefore we were limited by the amount of information collected in the original survey. We evaluated three levels of social support: individual, family, and neighborhood, which were the three levels of social support assessed by NSCH. Ideally, more degrees of social support could be assessed, and the amount of support in each category could be further characterized, for example by categorizing individual support by number of people in each parent’s daily support system. All types of support in this study were measures reported by parents, so it is possible that factors influencing the ways parents perceive their available social support could be impacting child screen-time, rather than actual differences in experienced support. Further studies might utilize scored questionnaires or interviews to quantify social support less subjectively. Additionally, we were limited by the inability to analyze certain co-variates influencing social support and/or screen-time, such as health literacy and neighborhood safety. Though we adjusted for several social factors shown to be associated with screen-time, our study did not evaluate all possible co-variates given the breadth of questions surveyed in the NSCH.

These findings, which have expanded upon and better characterized which types of social support are associated with optimal screen-time, may provide a necessary step in identifying possible routes for interventions to promote healthy screen-time in early childhood. This study suggests that interventions which strengthen parental support, such as community-based programs to support interpersonal needs, programs that strengthen support among families, and programs that encourage neighborhood networks, could facilitate healthy screen-time in children. Additionally, focusing on optimal screen-time in early childhood may have positive impacts on parental social networks and perceived support systems. This also suggests that when counseling on screen-time, pediatricians should recognize that feasibility of achieving optimal screen-time depends on contextual factors, such as parental social support.

## Conclusion

Overall, these findings show that multiple levels of social support at the individual, family, and community level are independently and jointly associated with optimal screen-time in early childhood, even when adjusting for socioeconomic factors. However, the mechanisms and directionality of these associations are still unclear. Given the well-known associations of screen-time with adverse outcomes, we reinvigorate the notion that social context can impact health outcomes starting as early as young childhood. In the light of screen-time recommendations that emphasize parental engagement with screens, this data can offer insight into factors that may encourage children to utilize screens in positive ways that promote social connection. Additionally, we suggest that lower child screen-time may enhance parental bandwidth, allowing for the fortification of social networks. Further studies should characterize degrees of social support, investigate exactly how the various types of social support are associated with screen-time, better characterize the role of parental stress in this relationship, and explore other confounding factors that influence screen-time. Our findings suggest that public health interventions which allocate resources to increasing individual, family, and neighborhood support with parenting, especially in low-income families, could have beneficial health effects on screen-time starting in early childhood. Our findings could also suggest that interventions aiming to reduce child screen-time would have positive impacts on developing parental support systems. Nevertheless, we show that pediatricians ought to ask about and carefully consider parental social context when holding screen-time conversations with patients and their families.

## Figures and Tables

**Table 1:** Study Demographics.

	Overall sample n = 19,741	Optimal Screen Time	
		Yes (n= 5,894)	No (n=13,405)
Assigned Sex			
Male	10,081 (51.07%)	2,960 (50.22%)	6,896 (51.44%)
Female	9,660 (48.93%)	2,934 (49.78%)	6,509 (48.56%)
Race and Ethnicity			
White, non-Hispanic	12,789 (64.78%)	4,041 (68.56%)	8,527 (63.61%)
Hispanic	3,014 (15.27%)	779 (13.22%)	2,146 (16.01%)
Black, non-Hispanic	1,001 (5.07%)	172 (2.92%)	782 (5.83%)
Other/Multi-racial, non-Hispanic	2,937 (14.88%)	902 (15.03%)	1,950 (14.55%)
Maternal Age			
Mean maternal age	31.12 (95% CI: 31.04–31.19)	31.90 (95% CI: 31.77–32.03)	30.81 (95% CI: 30.72–30.91)
Parental Education Level			
Less than high school	391 (1.98%)	130 (2.21%)	230 (1.72%)
High school degree or Equivalent	2,252 (11.41%)	486 (8.25%)	1,662 (12.40%)
Some college or technical school	3,767 (19.08%)	753 (12.78%)	2,913 (21.73%)
College degree or higher	13,331 (67.53%)	4,525 (76.77%)	8,600 (64.16%)
Parental Income			
0–99% Federal Poverty Level	2,347 (11.89%)	569 (9.65%)	1,670 (12.46%)
100%-199% Federal Poverty Level	2,972 (15.05%)	736 (12.49%)	2,140 (15.96%)
200%-399% Federal Poverty Level	5,710 (28.92%)	1,490 (25.28%)	4,125 (30.77%)
400% Federal Poverty Level or greater	8,712 (44.13%)	3,099 (52.58%)	5,470 (40.81%)
Main Variables			
Optimal screen-time	5,894 (29.86%)	-	-
Parental aggravation	924 (4.81%)	709 (5.32%)	214 (3.67%)
Individual support	16,437 (83.26%)	5,175 (88.42%)	11,190 (84.15%)
Family Support	16,577 (87.80%)	5,201 (90.88%)	11,284 (86.49%)
Neighborhood support	10,843 (54.92%)	3,517 (61.28%)	7,266 (55.68%)
Cumulative Support	8,892 (47.69%)	3,022 (53.30%)	5,833 (45.27%)

Weighted n= 21,470,296. Data from children aged 0–5 in the 2022 National Survey of Children’s Health. Screen-time was reported by parents. Optimal screen-time was defined as one hour or less daily. Individual support, neighborhood support, and family support were assessed in a self-reported survey. Chi squared models compared various study demographics in children with and without low screen-time.

**Table 2:** Logistic regression models for study predictors and low-screen time outcome.

Predictor	Unadjusted Odds Ratio	Adjusted Odds ratio, Low screen-time
Individual Support	1.46** (1.22, 1.75)	1.34* (1.11, 1.62)
Family Support	1.44*** (1.17, 1.76)	1.37** (1.10, 1.71)
Neighborhood Support	1.31** (1.16, 1.49)	1.21* (1.07, 1.38)
Cumulative support	2.39*** (1.56, 3.66)	2.24** (1.37, 3.67)

Logistic regressions modeled the association of various measures of parent social support including individual, family, neighborhood, or cumulative support, and one hour or less of screen-time daily controlling for race/ethnicity, maternal age, parental education, and poverty level. Models were weighted to represent the US population (weighted n=21,470,296).

* p<0.05

** p<0.01

*** p<0.001

**Table 3:** Logistic regression models for study predictors and low screen-time outcome stratified by income level.

Predictor	Low Income Families		Higher Income Families	
	Unadjusted	Adjusted	Unadjusted	Adjusted
Individual Support	1.18 (0.952, 1.47)	1.16 (0.913, 1.47)	2.12*** (1.61, 2.79)	2.12*** (1.61, 2.79)
Family Support	1.39* (1.07, 1.81)	1.40* (1.05, 1.88)	1.35 (0.981, 1.85)	1.29 (0.942, 1.75)
Neighborhood Support	1.45*** (1.25, 1.72)	1.39*** (1.17, 1.66)	0.944 (0.788, 1.13)	0.850 (0.711, 1.02)
Cumulative support	2.26** (1.38, 3.68)	2.46** (1.39, 4.33)	1.81 (0.880, 3.72)	1.62 (0.704, 3.74)

Data from children aged 0–5 in the 2022 National Survey of Children’s Health surveyed parents about screen-time and social support. Low screen-time was defined as one hour or less daily. Family income level was dichotomized to low-income (household income below 400% of the federal poverty level) and higher-income families (household income at or above 400% of the federal poverty level). Logistic regression analyses modeled the association of various types of social support: individual, family, neighborhood, or cumulative support with the outcome of low screen-time separately in low-income and higher-income families while controlling for maternal age, race/ethnicity, and parental education level. Analyses were weighted to represent the US population of young children.

* p<0.05

** p<0.01

*** p<0.001

## Highlights

- Individual, family, neighborhood, and cumulative support, as perceived by a caretaker, are independently associated with optimal early childhood screen-time.
- Family income moderates the protective effects of specific types of social support on early childhood screen-time.
- In families with low-income, family, neighborhood, and cumulative support are associated with optimal? early childhood screen-time.
- In families with higher-income, individual support is associated with optimal early childhood screen-time.
- Future studies may examine the role of parental stress and well-being in the relationship between parental social support and child screen-time.
